# Comparative study of debris and smear layer removal with EDTA and Er,Cr:YSGG laser

**DOI:** 10.4317/jced.54936

**Published:** 2018-06-01

**Authors:** Paloma Montero-Miralles, Daniel Torres-Lagares, Juan-Jose Segura-Egea, Maria-Ángeles Serrera-Figallo, Jose-Luis Gutierrez-Perez, Gabriel Castillo-Dali

**Affiliations:** 1 Department of Stomatology, School of Dentistry, University of Seville

## Abstract

**Background:**

To evaluate *in vitro*, the ability in removing debris and Smear Layer of 17% EDTA and Er,Cr:YSGG laser.

**Material and Methods:**

58 unirradicular teeth were instrumented with MTwo® and divided into 3 groups according to irrigation protocol: 17%EDTA, laser and a combination of 17%EDTA and laser. All samples were analyzed in the apical and middle third with Scanning Electron Microscope. The Chi-cuadrado and McNemar tests were used to determine the statistical analysis and data processing and analysis was performed with the statistical package StatGraphics Centurion XVI.

**Results:**

Debris analysis showed statistical significant differences when compared EDTA vs laser and EDTA vs EDTA+laser in the middle third. The Smear Layer removal showed statistical significant differences in the middle third when compared EDTA vs laser and EDTA vs EDTA+laser.

**Conclusions:**

Laser showed a greater cleaning capacity than EDTA in the middle third; the cleanliness was even better when combined laser with EDTA, so the effect is accumulative.

** Key words:**Root canal treatment, Smear Layer, Er,Cr:YSGG laser, debridement.

## Introduction

Smear Layer was described by McComb and Smith in 1975. It consists of a superficial layer in the root canal wall of 1-2 microns thickness (1) and contains organic and inorganic substances, microorganisms and necrotic debris (2). This Smear Layer could be infected and could protect bacteria in the dentinal tubules from irrigants (3), so its elimination is necessary prior to the root canal system obturation.

Many ways to remove the Smear Layer have been described, the most studied is chelants substances, like Acid Etilendiamintetrathetic (EDTA). Studies demonstrate that mechanical instrumentation along with chemical irrigants action do not achieve a complete removal of Smear Layer from the root canal walls (4,5), therefore we should use other techniques for this purpose. Laser is another method to eliminate the Smear Layer. High power lasers have been proposed to contribute in the conventional endodontic therapy for their capacity to eliminate microorganisms and increasing dentinal permeability by Smear Layer removal (6-9).

Er,Cr:YSGG laser has a 2980 nm wavelength and high absorption for water and hidroxypatatite (10). Several studies demonstrate its capacity to remove debris y Smear Layer after biomechanical instrumentation (11) and do not cause tissue carbonization or melting (11,12). This laser employed a laser beam pulsed source transmitted by a sapphire tip with air/water spray (13-15). When an interaction of laser energy with water and the target tissue occurred, this generated a tissue cut, creating a hydrokinetic system (13-15). The hydrokinetic debridement is a removal process of biological materials through a high speed water spray. During irradiation, water is heated and evaporates, resulting in a high pressure steam that causes a microexplosion of the dental tissues below its fusion point (16). Water vaporization into the mineral substrate causes an explosion of the surrounding material literally outwards (17).

Conventional tips are a limitation of this treatment. These tips produce a laser beam emission unidirectional towards apex, which complicates the access to the root canal wall with a laser. Fiber should move repeatedly in a spiral movement for all the canal walls during a sequence number to maximize the exposed area to the laser beam (18). Recently, a new tip has appeared, the Radial Firing Tips (RFT), which ends in a conical tip with an angle of 60 degrees to achieve a light expansion in a bigger cone, reaching all the root canal better, because it emits the light apical laterally (19).

Therefore, the objectives of the study was, firstly, to evaluate the capacity of removing debris and Smear Layer of 17% EDTA (considered as the Gold Standard) and Er,Cr:YSGG laser, and secondly, to verify if the combination of laser and a chelating agent improves this removal with the new Radial Firing Tips.

## Material and Methods

We selected a sample of 58 unirradicular teeth. Inclusion criteria were presence of one single canal, complete closed apex and no previous canal treatment. Exclusion criteria was presence of caries, calculus, open apex, radicular resorption, radicular fractures and two canals.

Samples were decoronated to obtain a homogeneity obtaining a final length of 16 mm. All samples presented apical patency and maintained it during all the treatment. A glide path was realized with K flexofile files (Dentsply-Maillefer, Tulsa, United States) to a 20 file and instrumentation was carried through the basic sequence of the rotatory system MTwo (VDW, Munich, Germany) with 10.04, 15.05, 20.06, 25.06 files and extending the sequence with the 30.05, 35.04 y 40.04 to one millimeter less than the total sample length, this is 5 mm. Each file was used in 10 teeth and discarded.

During all the procedure, we irrigated continuously with 1 ml of 4,2% sodium hypochlorite with a Monoject 3 ml syringe (Tyco HealthCare Group, Mansfield, USA) and needle (27g x1 1/4) situated at 1 mm less than working length, being changed between files. Once instrumentation was finished, the samples were kept in distilled water.

For the irrigation stage, samples were sealed in the apical area with wax, and a framework was constructed using the same material to create a reservoir for the irrigant.

• Group 1: Samples were irrigated for 1 minute with 5 ml 17% EDTA (Pulpdent, Oakland, USA), followed by 5 ml 4,2% sodium hypochlorite for 2 minutes and a final wash with 2,5 ml distilled water by a Monoject 3 ml syringe and needle (27g x1 1/4) situated one millimeter less than working length.

• Group 2: Samples were irradiated with Er,Cr:YSGG laser (BIOLASE®), with a 2.780 nm wavelength and a “Radial Firing Tips” RFT-2 , with a 275 microns diameter, making helicoidal movements from apical to coronal. A laser tip was introduced to 1 mm less than the working length, this is 14 mm. The activation of the laser lasted 5 cycles of 5 seconds each, with a break of 20 seconds beteen each cycle. A final wash with 2,5 ml distilled water was done by a Monoject 3 ml syringe and needle (27g x1 1/4) situated one millimeter less than working length.

• Group 3: Samples was irrigated for 1 minute with 5 ml 17% EDTA and irriadiated with Er,Cr:YSGG laser with the previous protocol and finally irrigated with 5 ml 4,2% sodium hypochlorite for 3 minutes and a final wash with 2,5 ml distilled water by a Monoject 3 ml syringe and needle (27g x1 1/4) situated one millimeter less than working length.

Samples were dried with 40 size paper points.

Er,Cr:YSGG laser was used following this parameter: 1.25 W, 50 Hz and 24% air and 30% water. Irrigant was placed with a Monoject 3 ml siringe and needle (27g x1 1/4) situated one millimeter less than working length for all the samples.

Samples were cut in mesio-distal direction for observation of the root canal with an ultra-fine diamond disc 20x0,25mm diameter and a low speed micromotor Volvere VMax (NSK, Japan. This cut does not penetrate into the canal lumen to avoid debris penetrating into the sample. With a chisel in the slot made with the disc and with a sharp blow the root was divided into 2 parts. Samples were prepared at room temperature to be watched under Scanning Electron Microscope. They are placed in a sputtering with a gold layer of 25 nm thickness (Emitech K550X). The microscope JEOL JSM-6400 as used with 20 kV and39 mm working length. Study areas were the apical and the middle third of the root canal. Images were obtained randomly at 0,5 mm and 5 mm from the apex at x500 and x1000.

Images were analyzed for an experimented and trained observer to view samples at SEM and to evaluate debris and Smear Layer in the dentinal surface. We used Hülsman modified classification to measure debris presence at x500 and Smear Layer presence at x1000 (20):

Value 0: Clear surface, all tubules open

Value 1: Most tubules are open, but debris remains

Value 2: Most tubules surface are covered

Value 3: All the surface is covered by debris

Association between the studied groups and cleaning scale were evaluated by chi-cuadrado test. For all tests a signification value *p*< 0,05 will be accepted. Frequency distribution was evaluated for each group in the results variable in its two determinations (500 y 1000) with related samples McNemar test. Processing and data analysis was carried out with the statistical package StatGraphics Centurion XVI.

## Results

-Comparative analysis of debris removal

Comparing group 1 and group 2, in the middle third we found statistical significant differences at x500 (*p*=0.0170). In both thirds, Er,Cr:YSGG laser group presented cleaner surfaces than 17% EDTA.

Comparing group 1 and group 3, in the middle third we found statistical significant differences at x500 (*p*=0.0042). In both thirds, a combination of Er,Cr:YSGG laser and 17% EDTA showed cleaner surfaces.

Comparing group 2 and group 3, we did not find statistical significant differences at x500, but in all cases, combination of Er,Cr:YSGG laser with 17% EDTA shower slightly better results of cleanliness.

-Comparative analysis of smear layer removal

Comparing group 1 and group 2, in the middle third, we found statistical significant differences at x1000 (*p*=0.0150). In both thirds, Er,Cr:YSGG laser group presented cleaner surfaces than 17% EDTA.

Comparing group 2 and group 3, in the middle third, we found statistical significant differences at x1000 (0.0051). In both thirds, combination of Er,Cr:YSGG laser and 17% EDTA showed cleaner surfaces.

**Figure 1:**
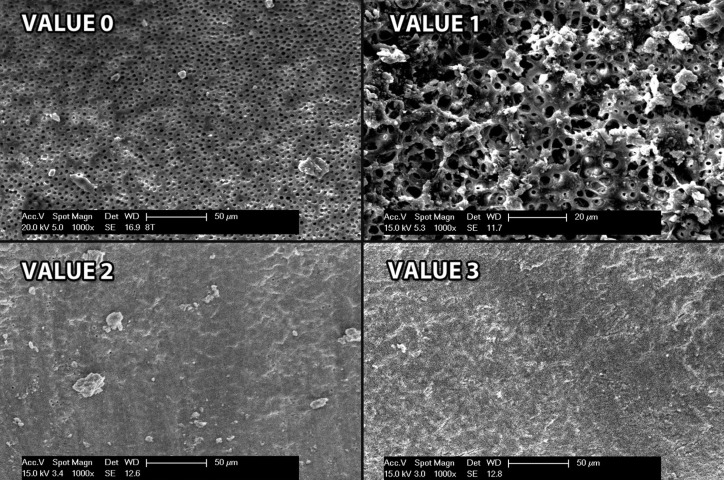
Images for observers.

**Figure 2:**
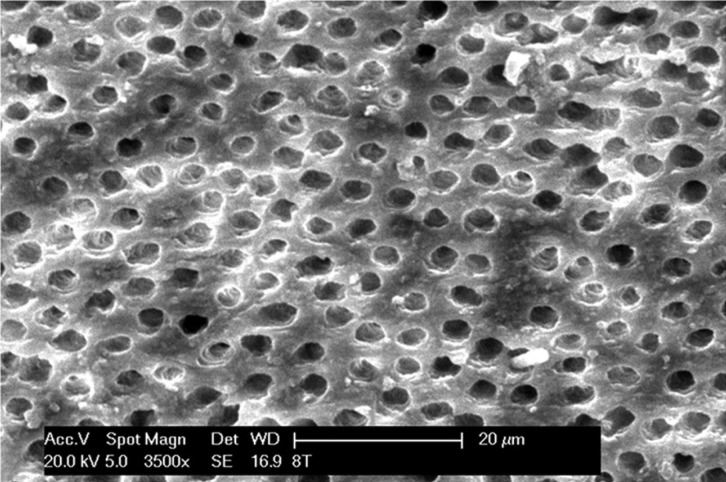
Detail of middle third in group 2 at x3500.

**Figure 3:**
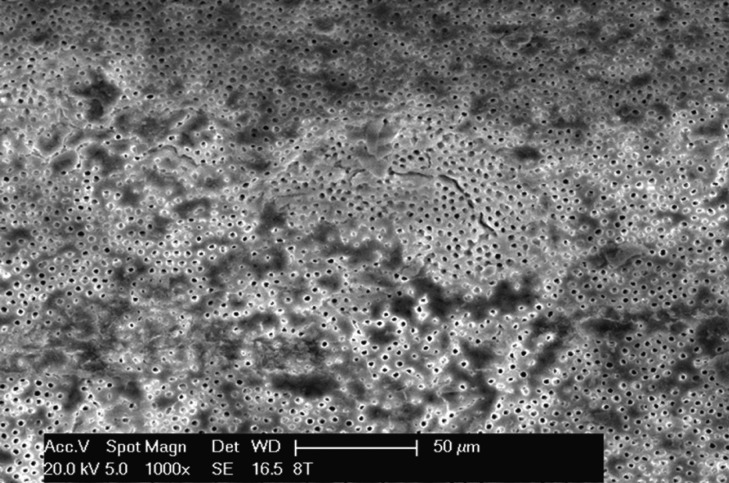
Detail of apical third in group 2 at x1000.

Comparing group 1 and group 3, we did not find statistical significant differences, but in both groups, a combination of Er,Cr:YSGG laser with 17% EDTA showed slightly better results of cleanliness, (Figs. 1-3,  Table 1).

**Table 1:**
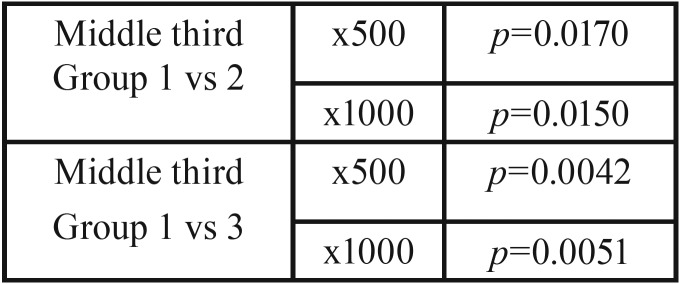
Significative groups.

## Discussion

Smear Layer removal has been studied by many authors, because of its presence in dentinal walls affected root canal system seal.

Laser inclusion in endodontics, supposed a new approach because of morphological changes in radicular surfaces, producing a debris and Smear Layer removal that allows dentinal tubules opening increasing therefore dentinal permeability (6-9).

In this study, we had wanted to verify the effect that laser produces in the root canal wall by Scanning Electron Microscope with regard to debris and Smear Layer removal being used alone or in combination with a chelating agent (17% EDTA).

In our study, when compared utilization of 17% EDTA in combination with Er,Cr:YSGG, results were always greater for Er,Cr:YSGG laser , which presented better cleanliness to 17% EDTA, these differences being statistically significant in the middle third in debris and Smear Layer analysis. These results agree with authors such as Biella-Silva (21), who studied apical, middle and coronal thirds using powers of 1.75W y 2.5W, they found Smear Layer free surfaces and opened dentinal tubules, but only statistical significant differences in the apical third at 2.5W, the cleanliness degree was better in the group that combined 17% EDTA and Er,Cr:YSGG laser. This difference could be due to the use of a 400 microns diameter fiber and a samples instrumentation of 60 size, so fiber had more space to move. George *et al.* (22), in their study got a Smear Layer removal degree in all groups where laser was used, the same as Moor (18), who found Smear Layer removal by “shock-waves” generation, with comparable results as passive ultrasonic irrigation. Ali *et al.* (23), in their results obtained no debris and Smear Layer in root canal walls at 2W in apical and coronal thirds, but they found many debris in the middle third, where the fiber makes more contact with the walls and a fused dentinal wall could be produced.

Other authors concluded that Er,Cr:YSGG laser utilization with water is effective in debris and Smear Layer removal, but these studies do not specify studied thirds, neither chelants or laser utilization. They only showed morphological changes during laser treatment at many different parameters (11,12,24).

Regarding the combination of chelants and Er,Cr:YSGG laser, in our study when comparing group 1 and group 3, group which combined 17% EDTA and Er,CrYSGG laser, this last one showed a better cleanliness but in a less evident way. Differences were statistically significant in debris and Smear Layer analysis in the middle third.

Therefore, we could accept the hypothesis that er,Cr:YSGG laser causes a mineralized tissue ablation and produced a dentinal tubules exposition and Smear Layer removal (25,26). The mechanisms of action of Er,Cr:YSGG laser is based on the expansion and implosion of the vapor lock with a secondary cavitation effect, which induces the movement of this fluid to a high velocity inside the root canal (27). This generates a combined effect, on one side the laser´s effect, and on the other hand because of chelants activation, in agreement with the results of our study. Scientific literature is capable to prove the hydrokinetic effect as a viable mechanism of laser ablation (28,13,14).

Apical third is the most complex zone, and although results are favorable to Er,Cr:YSGG, in this third, differences were not statistically significant.

The increase of temperature is a handicap on laser treatment, and we should avoid it using safety parameters that reach our purpose but safely. Eriksson *et al.* showed in their study that we should not increase in more than 10 degrees over the corporal temperature for more than a minute (28). Many authors showed that laser treatment with Er,Cr:YSGG is safety for perirradicular tissues and do not cause thermal damage, as many authors showed in their studies, finding an increase of 8 degrees, Ishizaki (12) using laser at 5W and Yamakazi (11) at 6W. This agrees with requirements proposed by Eriksson (28) and support the utilization of our parameters at 1.25W as a safety treatment.

Water utilization during laser treatment is very important to avoid undesirable effects. Morphological findings in other studies showed that irradiation without water produces an enamel and dentin carbonization, associated to an irregular structure and microdrills (6,11,26). Water plays an important role in hard tissues ablation in endodontic treatment (11), avoiding temperature increase and enhancing cutting efficacy (11,15,25).

It is essential to achieve a consensus regarding the laser parameters, as wavelengths or dosis, for treatment to be safe and effective.

## Conclusions

1. Regarding the middle third, Er,Cr:YSGG laser showed a better cleanliness with stadistically significative differences compared to 17% EDTA.

2. Regarding to laser and EDTA combination, this results in cleanliness áreas in the middle third, when compared to 17% EDTA, with stadistically significative differences.
